# Yttrium stabilization and Pt addition to Pd/ZrO_2_ catalyst for the oxidation of methane in the presence of ethylene and water†

**DOI:** 10.1039/d0ra10773e

**Published:** 2021-03-23

**Authors:** Hassnain Abbas Khan, Junyu Hao, Omar El Tall, Aamir Farooq

**Affiliations:** Clean Combustion Research Center, Physical Science and Engineering Division, King Abdullah University of Science and Technology (KAUST) Thuwal 23955-6900 Saudi Arabia hassnain.khan@kaust.edu.sa aamir.farooq@kaust.edu.sa; KAUST Core Labs, King Abdullah University of Science and Technology (KAUST) Thuwal 23955-6900 Saudi Arabia

## Abstract

Catalytic oxidation is the most efficient method of minimizing the emissions of harmful pollutants and greenhouse gases. In this study, ZrO_2_-supported Pd catalysts are investigated for the catalytic oxidation of methane and ethylene. Pd/Y_2_O_3_-stabilized ZrO_2_ (Pd/YSZ) catalysts show attractive catalytic activity for methane and ethylene oxidation. The ZrO_2_ support containing up to 8 mol% Y_2_O_3_ improves the water resistance and hydrothermal stability of the catalyst. All catalysts are characterized by X-ray diffraction (XRD), Brunauer–Emmett–Teller (BET), O_2_-temperature-programmed desorption (O_2_-TPD), and CO-chemisorption techniques. It shows that high Pd dispersion and Pd–PdO reciprocation on the Pd/YSZ catalyst results in relatively high stability. *In situ* diffuse reflectance infrared Fourier-transform (DRIFT) experiments are performed to study the reaction over the surface of the catalyst. Compared with bimetallic catalysts (Pd : Pt), the same amounts of Pd and Pt supported on ZrO_2_ and Y_2_O_3_-stabilized ZrO_2_ catalysts show enhanced activity for methane and ethylene oxidation, respectively. A mixed hydrocarbon feed, containing methane and ethylene, lowers the CH_4_ light-off temperature by approximately 80 °C. This shows that ethylene addition has a promotional effect on the light-off temperature of methane.

## Abstract 2

Addition of 8.0% Yttrium (Y) to ZrO_2_ substantially increased the activity and stability of Pd/ZrO_2_.

## Graphical abstract

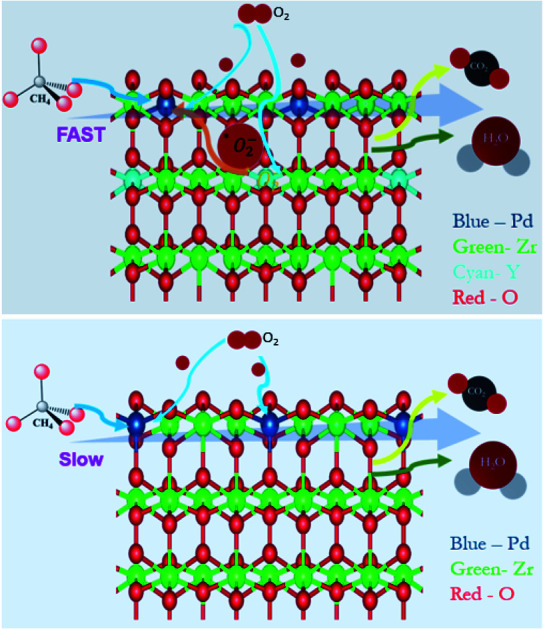

## Introduction

Hydrocarbons (HCs), such as methane (CH_4_) and ethylene (C_2_H_4_), are an environmental concern. Methane is the second major contributor to climate change.^1^ Furthermore, it has a radiative forcing efficiency that is approximately 120 times more than that of CO_2_.^2–4^ Environmental energy security directives are promoting the use of natural-gas vehicles because of the low value of C/H compared to that of gasoline implies lower CO_2_ emissions.^5^ However it contributes significantly to the release of unburned methane besides fugitive emissions.^6^ Accordingly, converting methane to a less potent greenhouse gas, along with overall emission reduction, is necessary. Compared to collecting and storing methane, catalytically converting it to carbon dioxide is a thermodynamically favorable reaction. The largest activation barrier is associated with the dissociation of strong C–H bonds (435 kJ mol^−1^), and catalysts can play a crucial role in overcoming this barrier.^7^CH_4_ + 2O_2_ → CO_2_ + 2H_2_O; Δ*H*_r_ = −803 kJ mol^−1^.

Ethylene is one of the most important intermediates of the hydrocarbon combustion process. It is a harmful pollutant that is emitted into the environment because of the incomplete combustion in power plants and vehicles. Similar to methane, ethylene can be converted to carbon dioxide over a catalyst.^8^C_2_H_4_ + 3O_2_ → 2CO_2_ + 2H_2_O; Δ*H*_r_ = −1322 kJ mol^−1^.

Pd and Pt are regarded as highly stable catalysts for the total combustion of CH_4_ and C_2_H_4_, respectively.^9,10^ However, the deactivation due to sintering at high reaction temperature (400–600 °C) and wet (H_2_O) feeds remains a challenge.^9,10^ Attempts have been made to resolve catalyst deactivation by adding Pt to Pd-based monometallic catalysts.^11–14^ Nevertheless, the influence of Pt on the improvement of bimetallic Pd–Pt catalysts is inconclusive. Though some literature state that alloying Pt to Pd increases catalytic activities,^8,12,15,16^ others argue that the impact of adding Pt to Pd is not obvious.^14,17,18^ Persson *et al.* compared the catalytic activity of Pd and bimetallic Pt–Pd catalysts and found that the addition of Pt to Pd has detrimental effects and it even lowers the performance of the monometallic Pd catalyst.^14^ In another study by Strobel *et al.*, the effects of different molar ratios of Pt to Pd were compared. It was observed that higher Pt molar ratios have adverse effects on the activity of Pd catalysts.^17^ However, notably, the addition of Pt has promotional effects on the dispersion of supported Pd catalysts.^8^

Among other factors, support materials have an important influence on the catalytic properties. The influence of the support originates from its interaction with the depositing metal species. The dispersion, oxidation states, thermal stabilities, and oxygen mobility are governed by the nature of the support materials.^19–23^ These characteristics directly influence the catalytic performance of the active novel metal. Several studies have been conducted on Pd supported on Al_2_O_3_, TiO_2_, CeO_2_, SnO_2_, MgO, zeolites,^24,25^ SiO_2_,^26^ and modified Al_2_O_3_–TiO_2_ (ref. 27) catalysts. CH_4_ combustion follows the Mars–Van Krevelen mechanism over supported Pd catalysts.^28^ Therefore, the rate of release of active oxygen species by the support materials and their replenishment could affect the catalytic activity for CH_4_ combustion.

Yoshida *et al.* reported that the Pd on moderately acidic oxides, such as ZrO_2_ and Al_2_O_3_, is more acidic than the Pd supported on strong acidic or basic oxides.^29^ ZrO_2_ has more surface oxygen vacancies and relatively high oxygen ion conductivity, which help maintain the PdO states during the reaction.^30,31^ Therefore, several reports claim that Pd/ZrO_2_ shows better catalytic performances in wet and dry conditions, compared to Pd supported on Al_2_O_3_ support.^32,33^ CeO_2_ has a relatively high ability to store and release oxygen, and the addition of CeO_2_ to ZrO_2_ leads to an increase in activity, though the catalysts are severally deactivated at low temperatures in real exhaust conditions where moisture is significantly high.^26^

Owing to the low temperature of vehicle exhausts during cold starts, it is challenging to effectively oxidize hydrocarbon emissions. Therefore, it is desirable to have good catalytic materials, which show activity at low temperatures. Pd/ZrO_2_ has the potential to meet this challenge.^22,32,34–41^ It is generally accepted that PdO–Pd is active for the combustion of CH_4_.^14^ ZrO_2_ offers oxygen exchange for Pd–PdO during the CH_4_ oxidation, and the lattice oxygen transport by ZrO_2_ greatly contributes to the oxygen pool of the supported PdO.^23,42,43^ ZrO_2_ has different phases, which also influence the catalytic performance.^34,36,43^ There is no clear information available whether the Pd supported on the monoclinic phase of ZrO_2_ is an active or tetragonal phase. However, it has been communicated in previous reports that the tetragonal phase of ZrO_2_ is more active for CH_4_ combustion with relatively high hydrothermal stability.^41^ Additionally, the phase transition in ZrO_2_ during the reaction hinders the performance and leads to the sintering and burial of Pd metal.^44^ The metastable tetragonal ZrO_2_ phase with high surface area shows promising combustion properties, and the catalyst remains stable during the test duration.^33,45^

Y_2_O_3_ (excellent O^−2^ conductor)-stabilized ZrO_2_(YSZ) is an increasingly significant catalyst carrier. Adding a specific amount of an oxide, such as Y_2_O_3_, to ZrO_2_ stabilizes the tetragonal phase of ZrO_2_ and improves its properties.^46^

In this work, Pd, Pt, and their alloys are supported on commercial ZrO_2_ and YSZ support materials to study their performance for HC (CH_4_ and C_2_H_4_) oxidation. Furthermore, the water resistance and hydrothermal stabilities of the prepared catalysts are investigated. The effect of dopants on the structural and redox properties is elucidated by characterizing the Mx/ZrO_2_ and Mx/YSZ (8 mol% Y_2_O_3_–ZrO_2_) catalysts. Our study will help to understand the influence of promoters on the performance of Pd/ZrO_2_ catalysts. This will help design for commercial/practical applications.

## Experimental

### Materials

Zirconium(iv) oxide powder (5 μm) and chloroplatinic acid hexahydrate were acquired from Sigma-Aldrich. Tetraaminepalladium(ii) chloride monohydrate was supplied by Alfa Aesar. YSZ (TZ-8Y) was purchased from Tosoh Corporation, Japan. Millipore deionized (DI) water was used as the solvent during catalyst synthesis.

### Catalyst synthesis

The wet impregnation method was used to synthesize all the catalysts. Briefly, 1.0 g of the support materials (YSZ and ZrO_2_) was vacuum dried for 12 h to remove moisture and clean the pores. The dried support was dispersed in 180 mL of DI water and sonicated for 60 min at 25 °C. To the sonicated dispersion, 20 mL of an aqueous solution of the metal salt (1 wt% metal loading) was added dropwise. The resulting suspension was mounted onto a rotary evaporator and rotated for 3 h to gently mix the support and salt solution. The solvent was desiccated under reduced pressure at temperature close to the boiling point of water (80 °C). The powder sample was collected and dried in a vacuum oven at 120 °C for the complete removal of the solvent in the pores. The dried sample was calcined in a muffle furnace at 600 °C in air for 3 h. The temperature was ramped up at 2 °C min^−1^. The Pd–Pt bimetallic catalysts were synthesized by the same method, keeping the overall metal loading at 1.0 wt% (0.5 Pd : 05 Pt).

### Catalyst characterization

Catalyst characterization details and procedures are given in the ESI† file.

### Activity measurement

A U-shaped quartz reactor with an inner diameter of 4 mm was used to test the performance of the catalysts in CH_4_ and C_2_H_4_ oxidation. The powdered catalyst was pressed to uniform-sized pellets and sieved to 45–60 μm. The reactor was heated in an electric furnace using an advanced temperature controller. A thermocouple (K-type) was placed inside the reactor, touching the catalyst bed, to record the real-time temperature. Gas-phase reactions without the catalyst bed inside the reactor were performed in the temperature range of 200–700 °C, and no significant conversions were observed. The composition of the feed gases was maintained at 1.0 vol% CH_4_/20% O_2_ diluted with nitrogen to maintain the total flow of 33.33 mL min^−1^. Subsequently, 25 mg of the dilute catalyst was placed inside a reactor using quartz wool, which corresponds to a GHSV of 80 000 mL g_cat_^−1^ h^−1^. The residence time was varied by increasing the flow rates of the feed gases and decreasing the weight of the catalyst. All the reactions were performed at 1 atm pressure. All the reactant and product gases were analyzed using an online gas chromatograph equipped with a thermal conductivity detector (TCD) and flame ionization detector (FID). The conversion activities of the catalyst were calculated as follows:
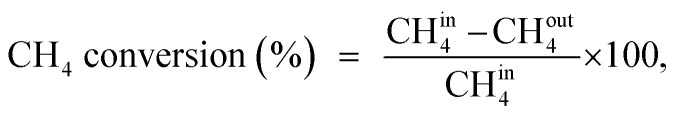

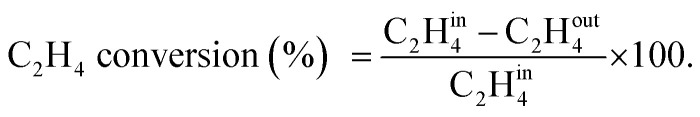


## Results and discussion

### Catalytic performance

#### Catalytic performance for methane oxidation

**Fig. 1:**
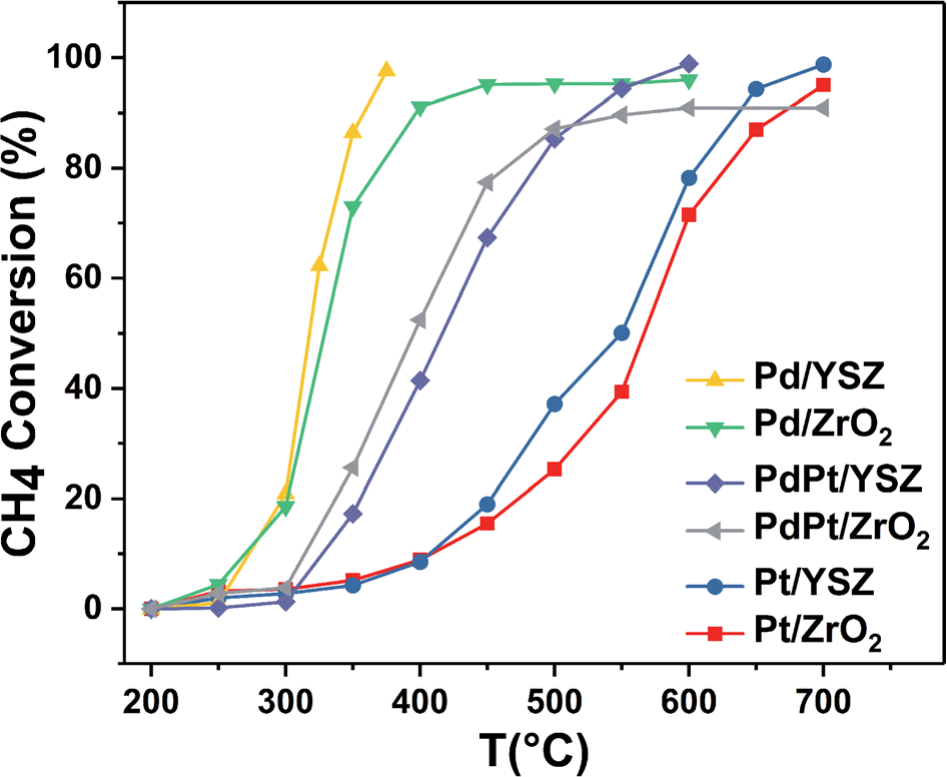
Light-off temperature profiles of CH_4_ oxidation over the synthesized catalysts (mass of catalyst: 0.025 g; total flow rate: 33.33 mL min^−1^; 1 volume% CH_4_ in 20% O_2_, balance N_2_; GHSV: 80 000 mL g_cat_^−1^ h^−1^).

Fig. 1 shows the light-off temperature profiles of all the prepared catalysts for the standard dry methane feed conditions. The light-off temperatures (*T*_10%_) of Pd/ZrO_2_, Pd/YSZ, Pt/ZrO_2_, Pt/YSZ, PdPt/ZrO_2_, and PdPt/YSZ were 276 °C, 275 °C, 405 °C, 405 °C, 310 °C, and 330 °C, respectively. The light-off temperatures recorded for all the catalysts are reported in Table 1. Bimetallic catalysts prepared with equal ratios (0.5 Pd : 0.5 Pt) were found to be less active than the reference monometallic Pd catalyst. Pt supported on ZrO_2_ was the least active and could not achieve 100% conversion even at 700 °C. YSZ-supported catalysts did not show any promotional effect in lowering the light-off temperature. However, relatively high activity was recorded when the temperature was increased. Significantly, 100% conversion was achieved at 360 °C. The methane conversion is directly relevant to the intrinsic reaction kinetics and mass transfer. Experimentally, it was validated that methane conversion presented an inverse proportion to the space velocity, and the results are presented in Fig. S1.† The steady state conversion rate can be achieved at a certain temperature with increasing flow rate. A further increase in flow rate will have little effect on the conversion of methane. At higher flow rates the mass transfer limitations thus become negligible.^47^

**Table 1:** Conversion temperatures (°C) for HC oxidation over the prepared catalysts

Catalyst	Conversion								
	Methane			Ethylene			Mix-feed^a^		
	*T*_10%_	*T*_50%_	*T*_90%_	*T*_10%_	*T*_50%_	*T*_90%_	*T*_10%_	*T*_50%_	*T*_90%_
Pd/ZrO_2_	276	330	422	224	270	325	267	326	443
Pd/YSZ	275	315	354	210	260	324	190	311	340
PdPt/ZrO_2_	310	392	600	103	160	275	300	380	600
PdPt/YSZ	330	415	527	76	118	227	307	403	450
Pt/ZrO_2_	405	568	675	53	88	173	400	545	700
Pt/YSZ	405	550	645	50	76	110	350	540	700

a Mixed feed gas composed of 1.0% of CH_4_ and 1000 ppm of C_2_H_4_, 20% of O_2_, balance N_2_.

#### Catalytic performance for ethylene combustion

**Fig. 2:**
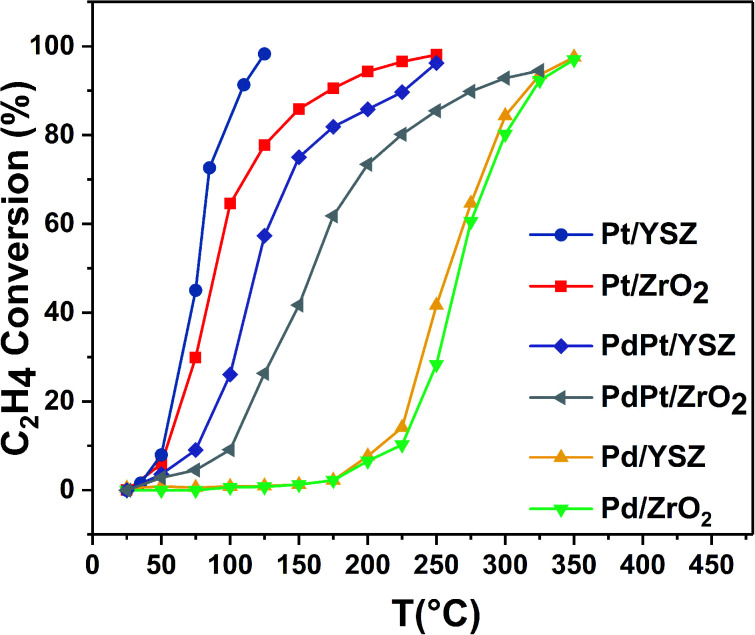
Light-off temperature profile of C_2_H_4_ oxidation over the synthesized catalysts (mass of catalyst: 0.025 g; total flow rate: 33.33 mL min^−1^; 1000 ppm C_2_H_4_/22% O_2_ in balance Ar; GHSV: 80 000 mL g_cat_^−1^ h^−1^).

An initial gas concentration of 1000 ppm was used to study the combustion of C_2_H_4_ over Pd, Pt, and PdPt catalysts on YSZ and ZrO_2_ supports. Table 1 presents the *T*_10_, *T*_50_, and *T*_90_ temperatures. As expected, the activity trends shifted inversely to CH_4_ oxidation on the prepared catalysts, as shown in Fig. 2. Monometallic Pt on YSZ was highly active for C_2_H_4_ oxidation. Complete oxidation was achieved at a low temperature of 125 °C. Consistent with previous reports, ZrO_2_- and YSZ-supported Pd were the least active. However, the Y_2_O_3_ promotional effect was prominent, and Pd supported on YSZ showed better catalytic performance as compared to Pd/ZrO_2_. The weakly supported Pd and Pt catalytic performance for C_2_H_4_ and CH_4_, respectively, have been extensively reported for lean conditions. To improve the performance, bimetallic catalytic systems are preferred for the oxidation of mixed hydrocarbon feeds.

#### Catalytic performance for mixed hydrocarbon feed combustion

**Fig. 3:**
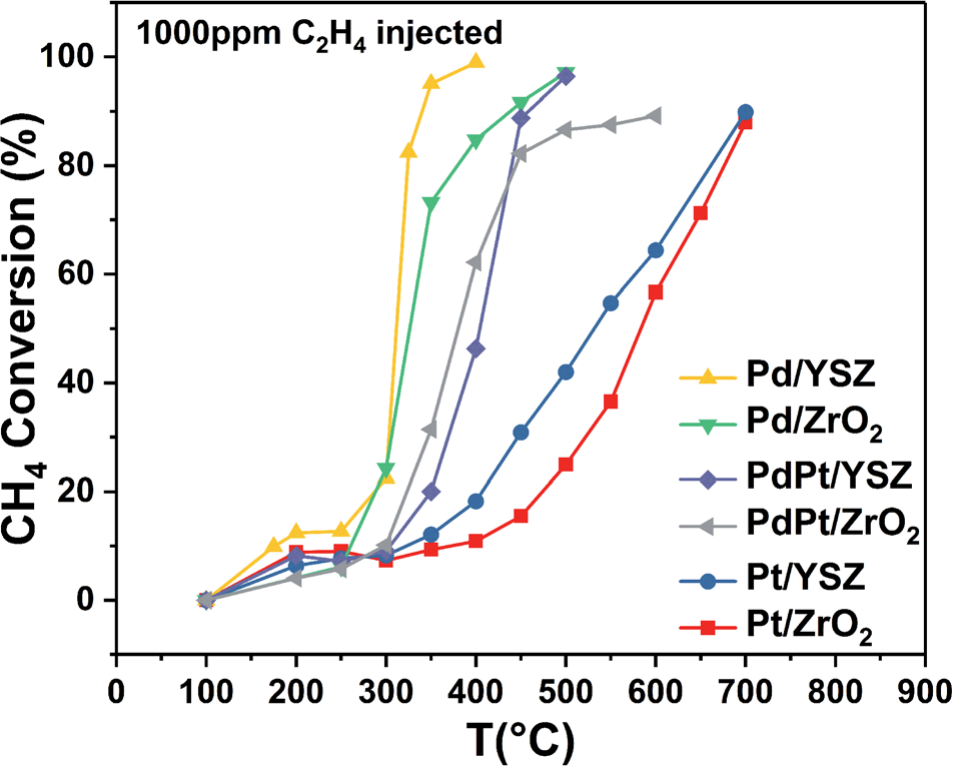
Conversion profiles for CH_4_ oxidation over the synthesized catalysts (mass of catalyst: 0.025 g; total flow rate: 33.33 mL min^−1^; 1 volume% CH_4_ in N_2_, 1000 ppm C_2_H_4_, 20% O_2_; GHSV: 80 000 mL g_cat_^−1^ h^−1^).

Exhaust gas is composed of a wide range of hydrocarbons; however, two of the major constituents with high heat sink are methane and ethylene. Therefore, the co-oxidation of CH_4_ and C_2_H_4_ was studied on all the prepared catalysts by adding 1000 ppm of ethylene/Argon as diluter gas, which is quantitatively 0.03% of the total fuel (1.0 vol% CH_4_) in feed gas. The low concentration of C_2_H_4_ was fully converted <99% below 160 °C. Fig. 3 shows the light-off temperature curves for CH_4_ with low concentration of C_2_H_4_. Notably, the light-off temperature of pure CH_4_ was significantly shifted to relatively low temperatures for the Pd/YSZ catalyst. The Pd/ZrO_2_ catalyst showed no prominent effect. However, a slight increase in *T*_90%_ temperature was evident. This may be due to the deactivation of Pd/ZrO_2_. This deactivation may be ascribed to the reaction of water and sintering due to high temperature. In the presence of C_2_H_4_, the CH_4_ light-off temperature shifted to relatively low temperatures over YSZ-supported Pd, Pt, and Pd–Pt. It was recorded that the *T*_10%_ of CH_4_ reduced to 30 °C on a scale in the presence of C_2_H_4_. This superior conversion at low temperatures can be explained by the exothermicity of ethylene oxidation. The C_2_H_4_ oxidation to CO_2_ was completed when CH_4_ oxidation began below 180 °C. C_2_H_4_ locally increased the catalyst bed temperature; thus, the CH_4_ was activated at a low temperature. At relatively high temperatures, the reaction rates were controlled by methane combustion, and, therefore, no prominent effect was observed in the presence of C_2_H_4_.

### Active surface area

Metal dispersion is typically determined using H_2_, O_2_, or CO chemisorption because these methods are commonly available and can be applied to highly diluted catalysts.^48^Table 2 shows the dispersion and metal surface area of the prepared catalysts. X-ray fluorescence spectroscopy (XRF) was used to quantify the impregnated metal components on all the prepared catalysts. The metal dispersion, specific metal surface area and average particle size of Pd, Pt and PtPd were measured by CO chemisorption method. The metal dispersions were: Pd/YSZ(6.73), Pd/ZrO_2_(6.37), Pt/YSZ(9.71), Pt/ZrO_2_(5.4), PtPd/YSZ(12.2), and PtPd/ZrO_2_(4.27). The results showed that metals were well dispersed on the support surface during the impregnation step. Y_2_O_3_ addition to ZrO_2_ increased the dispersion. It was noted that all the metals deposited on the YSZ supports were smaller than those on the ZrO_2_ supports. Particle size influenced the rate of reaction, and, for dispersed catalysts, the rate of reaction was proportional to the exposed active sites. The catalytic activities of the prepared catalysts were in good agreement with the dispersion of Pd, Pt, and Pd–Pt supported on YSZ and ZrO_2_.

**Table 2:** CO chemisorption and XRF quantification for the pristine catalysts

Catalyst (pristine)	CO adsorption (cm^3^ g^−1^)	Dispersion (%)	Metal Sa (m^2^ g^−1^)	Particle size (nm)	Metal percentage (%)
Pd/ZrO_2_	0.13	6.37	0.28	14.7	1.01
Pd/YSZ	0.14	6.73	0.30	12.9	0.98
Pt/ZrO_2_	0.06	5.4	0.13	17.47	0.98
Pt/YSZ	0.12	9.71	0.26	9.72	1.10
PdPt/ZrO_2_	0.09	4.27	0.19	26.2	0.4 : 0.52
PdPt/YSZ	0.20	12.2	0.42	7.6861	0.50 : 0.50

### Phase identification by XRD

**Fig. 4:**
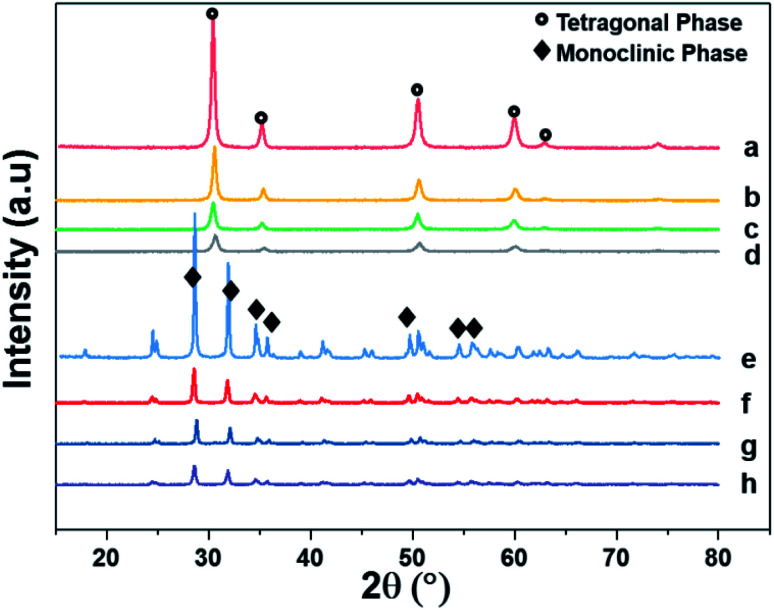
X-ray diffraction patterns of supports and pristine catalysts calcined at 600 °C for 3 h in air. (a) YSZ (b) Pd/YSZ (c) Pt/YSZ (d) PdPt/YSZ (e) ZrO_2_ (f) Pd/ZrO_2_ (g) PdPt/ZrO_2_ and (h) Pt/ZrO_2_.

**Fig. 5:**
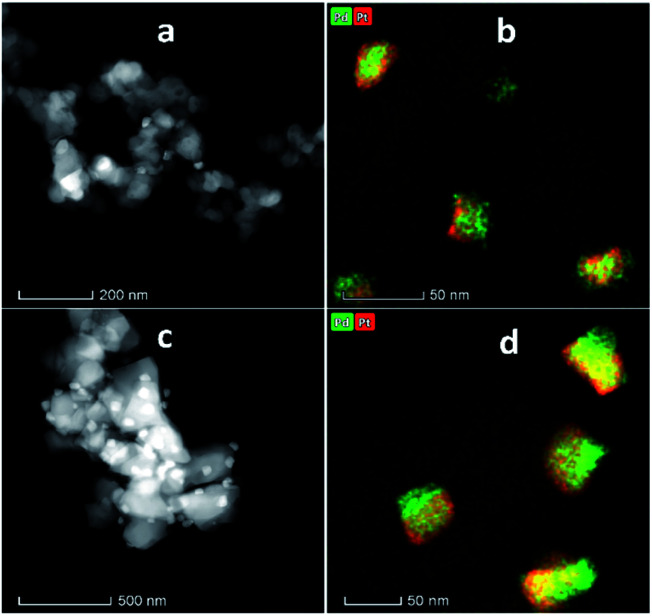
STEM and EDX elemental mapping (a and b) PdPt/YSZ, (c and d) PdPt/ZrO_2_.

Fig. 4 shows the XRD patterns of the support and pristine catalysts. YSZ exhibited the tetragonal phase of pure zirconia. No evidence of Y_2_O_3_ was observed by XRD. Fig. 4(a–d) shows the diffraction patterns for the YSZ support and Pd, Pt, and Pd–Pt supported on the YSZ catalysts. The peaks at 30.2 [101], 50.2 [112], and 60.2 [211] correspond to the blueprints of the tetragonal phase of ZrO_2_ in the joint committee on powder diffraction standards (JCPDS). The pristine and spent catalyst (see Fig. S2†) XRD patterns did not show any peaks for Pd and Pt metals. Fig. 4(e and f) presents the diffraction patterns for the ZrO_2_ support and Pd, Pt, and Pd–Pt supported on the ZrO_2_ catalysts. The ZrO_2_ support and catalysts only presented the monoclinic phase. The peaks at 24.6, 28.6 (111), and 31.9 (111) correspond to JCPDS card no. [JCPDS 37-1484], which was assigned to the pure monoclinic phase of ZrO_2_. There was no evidence of Pd, Pt, or alloy detected in the XRD patterns. This indicated that the impregnated metals were uniformly dispersed in the support macro and microporous matrix. Another possible reason was the low weight percentage of Pd and Pt in the catalyst. However, the presence of Pt and Pd was confirmed by XRF and TEM EDX color mapping (shown in Fig. 5). It was confirmed that Pt and Pd were present in the alloyed form. No evidence of individual metal was found in the TEM scan.

The surface areas of the pristine and spent catalyst were measured by the Brunauer–Emmett–Teller (BET) method using krypton and nitrogen as adsorbate gases; the results are given in Table S1.† As shown in Fig. S3,† the N_2_-isotherms for ZrO_2_ and Pd, Pt, and Pd–Pt supported catalysts showed that the samples exhibited type-IV isotherms with H4 hysteresis loops in the relative pressure range of 0.5–1.0 *P*/*P*_o_, indicating mesoporous (intraparticle) structures. The YSZ support showed type-II adsorption–desorption isotherms, indicating that YSZ had a mesoporous structure. It also showed hysteresis (H3); however, the disordered desorption isotherm showed that the pore structure was not well defined or uniform. The pore size distribution calculated by the Barrett–Joyner–Halenda (BJH) method was 12.1 nm. The BET surface area of the YSZ support was 9.0 m^2^ g^−1^. After the impregnation and calcination at 600 °C, the surface area did not change significantly. Notably, the pores were mesopores and the intraparticle spaces were void, reducing the diffusion barriers when the reaction was performed. As the combustion reaction generates moisture, CO_2_, during the reaction, catalysts with fewer diffusion limitations provide rapid transport of reactants and products. Similar behaviors were reported for the Pd/ZrO_2_ catalysts, where the catalysts with relatively high pore diameters of the support materials displayed better performance.^33,49^

### O_2_-TPD analysis

**Fig. 6:**
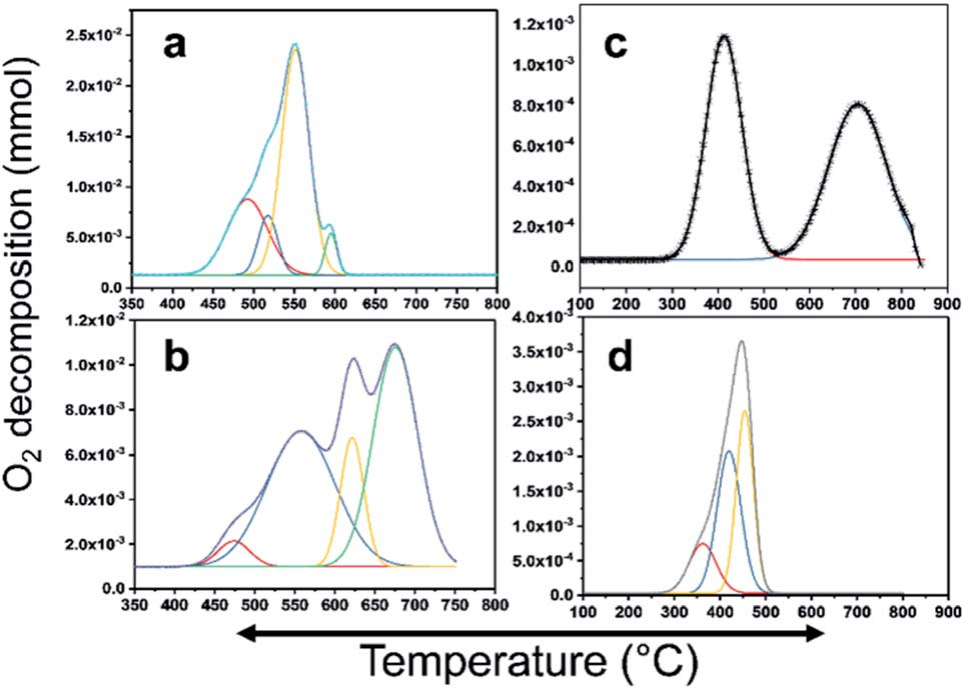
PdO decomposition analysis results using O_2_-TPD. (a) Pd/YSZ, (b) Pd/ZrO_2_, (c) PdPt/YSZ, and (d) PdPt/ZrO_2_.

CH_4_ oxidation is strongly reliant on the oxidation state of the active metal in the catalyst. There are many studies on the active phase of the metal for C–H bond activation. There is a consensus that PdO is highly active. In this study, PdO was closely investigated, where Pd–PdO and Pt–PtO decomposition and CH_4_ oxidation were compared. To completely understand the CH_4_ oxidation, O_2_-temperature-programmed desorption (O_2_-TPD) analysis was conducted. The products were analyzed using a TCD and an in-line quadrupole mass spectrometer. The TCD signals were matched with the MS signals to confirm the species for quantitative analysis. The O_2_-TPD signals were deconvoluted to determine the different temperatures at which the PdO species decomposed to metallic Pd, as shown in Fig. 6. Notably, multiple PdO decomposition temperatures could be resolved. As shown in Fig. 6(a), the Pd/YSZ catalyst showed the highest quantities of the PdO species. The initial peak was observed at 492 °C, and there was a high-intensity peak at 594 °C with small shoulders at 517 °C and 551 °C. Fig. 6(b) shows low-intensity peaks at 471 °C, 555 °C, 621 °C, and 625 °C for Pd/ZrO_2_. As shown in Fig. 6(c), Pd–Pt/YSZ showed peaks at 413 °C and 703 °C. Pt–Pd/ZrO_2_ showed a considerably low-intensity peak at 361 °C and two peaks at 417 °C and 453 °C. No evidence of oxygen from PtO decomposition was observed in the TCD and MS signals in the temperature range of 25–850 °C. Therefore, it may be concluded that CH_4_ oxidation is more favored for PdO states and Pt metal is the active state for C_2_H_4_ oxidation. As the temperature increased, Pd started to provide active sites for the C_2_H_4_ activation, and similarly, Pt metal started to partially oxidize and behave as an active component for the CH_4_ oxidation. Generally, Pt and Pd alloy resist the formation of oxides, but with increasing temperature the metals start to segregate and some PdO–Pd or PdO–Pt sites emerge, which can activate the C–H bond and result in the oxidation of CH_4_ to CO_2_. Most of the catalytic processes are redox or electron transfer processes, where the metal–substrate has to act as a donor–acceptor couple, and the metals can accept or donate electrons to form intermediate radicals that quickly form products. The redox properties of the ZrO_2_ support increased significantly when it was doped with Y_2_O_3_, resulting in more PdO species with varying bond strengths. Overall, this doping resulted in the release of more oxygen from the Pd catalyst.

### *In situ* DRIFT analysis

**Fig. 7:**
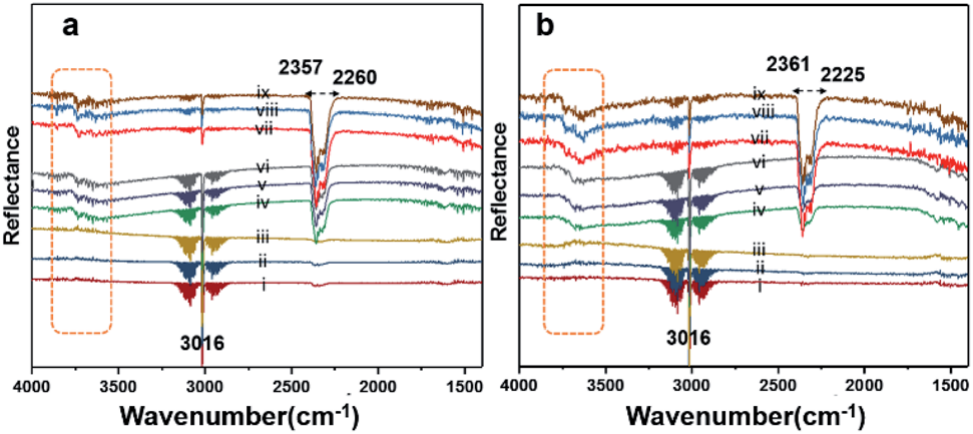
DRIFT spectra for (a) Pd/YSZ and (b) Pd/ZrO_2_. The analyses were performed at a flow of 2% CH_4_/N_2_ and O_2_ (20 vol%) at temperatures of 250 °C (i–iii), 350 °C (iv–vi), and 550 °C (vii–ix) in a continuous flow (33.33 mL min^−1^) heated cell.

Diffuse reflectance infrared Fourier-transform (DRIFT) analysis was performed using a Thermo Nicolet 6700 Fourier-transform infrared (FTIR) machine in diffusive reflectance mode. The FTIR machine was mounted with a high-temperature stainless steel reaction cell. The sample cell was covered with a dome-like cap, which had KBr windows and a liquid nitrogen-cooled detector. The DRIFT analysis was conducted using wavenumbers in the range of 800–4000 cm^−1^ at the spectral resolution of 4 cm^−1^. To measure the accurate temperature, a K-type thermocouple was placed inside the sample holder. MKS mass flow controllers were connected for the controlled flow of all the gases. All the catalyst samples were degassed and preheated before the analysis. The same feed gas compositions were used during the DRIFT analysis and in the quartz reactor for comparison purposes. Fig. 7(a) shows the spectrum for Pd/YSZ, and Fig. 7(b) shows the spectrum for the Pd/ZrO_2_ catalyst. The broad peaks with doubleheaders at 2225–2360 cm^−1^ were assigned to CO_2_. The sharp peak at 3016 cm^−1^ corresponded to CH_4_. The area highlighted with dotted rectangle was assigned to the hydroxyl region, which ranged from 3200 to 3800 cm^−1^. It was difficult to resolve the fine IR signals for OH groups because of the inferior signal-to-noise ratio on YSZ and ZrO_2_ supported on active metals (Pd, Pt). The small low-intensity signal at 3734 cm^−1^ may represent the Pd–OH masked by the OH signals of the YSZ carrier of Pd. Pd/ZrO_2_ presented broad signals in the 3200–3800 cm^−1^ region. A similar study was conducted to investigate the OH peaks on Pd supported on Al_2_O_3_. Signals at six wavenumbers were recorded. The signals at 3770, 3723, and 3680 cm^−1^ were allocated to hydroxyl on the support materials (Al_2_O_3_) and the three signals at wavenumbers 3556, 3697, and 3733 cm^−1^ were attributed to OH on PdO.^50^ Ciuparu *et al.* also confirmed the same wavenumbers assigned to OH signals associated with metal oxides.^51^ The signals at 3634 and 3628 cm^−1^ were assigned to terminal OH species that are attached to a single atom.^14^

**Fig. 8:**
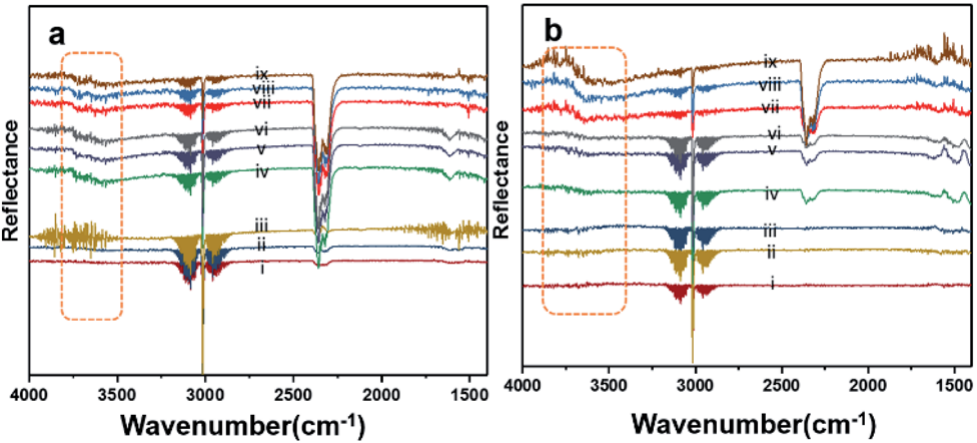
DRIFT spectra for (a) Pd–Pt/YSZ and (b) PdPt/ZrO_2_. The analyses were carried out at a flow (33.33 mL min^−1^) of 2% CH_4_/N_2_ and O_2_ (20 vol%) at temperatures of 250 °C (i, ii, iii), 350 °C (iv, v, vi), and 550 °C (vii, viii, ix) in a heated cell.

Fig. 8 shows the results of the DRIFT analysis for the Pd–Pt catalysts supported on YSZ and ZrO_2_. OH accumulation was minimized in the alloy catalysts on both the supports. Nevertheless, the alloy catalyst did not show superior catalytic performance to Pd supported on YSZ or ZrO_2_. The –OH groups that can poison the catalyst *via* the formation of Pd(OH)_2_ were mainly derived from the reaction. Pd supported on YSZ was resistant to OH accumulation. However, the ZrO_2_-supported catalyst showed gradual increases in –OH intensity, which was considered to be the reason for their deactivation in relatively long-duration reactions.

### Cyclic stability test

**Fig. 9:**
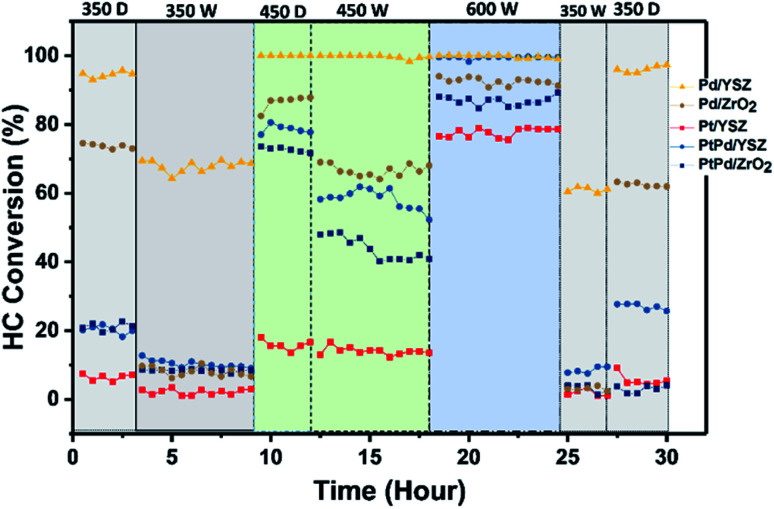
Cyclic stability test results for the prepared catalysts under dry and wet (12 vol% H_2_O) conditions. Feed composition: 1.0 vol% CH_4_, 1000 ppm C_2_H_4_, 22% O_2_ diluted with nitrogen at a GHSV of 80 000 mL g_cat_^−1^ h^−1^.

To validate the promotional effect of Y on ZrO_2_ and Pt on Pd toward water resistance and deactivation prevention, experiments in cyclic (dry ↔ wet) patterns were performed. The reaction time was prolonged up to 30 h at a GHSV of 80 000 mL g_cat_^−1^ h^−1^ in a continuous run. Fig. 9 shows the time on stream activity data for 1% CH_4_/1000 ppm C_2_H_4_ combustion. To generate wet conditions, 12 vol% water in the form of steam was added along with the HCs for these experiments. As seen in Fig. 9, the catalytic activities of the catalysts supported on ZrO_2_ were progressively degraded when the reaction environment was switched from dry to wet feed conditions. The Pd catalyst supported on YSZ showed promising activity throughout the cycling, and no prominent degradation in activity was observed for both reaction environments. The Pd–Pt catalyst supported on both YSZ and ZrO_2_ showed a steep decrease in activity. This showed that the addition of Pt at an equal ratio to Pd did not inhibit the water effect since Pt is very sensitive to water at relatively low temperatures. In this study, the deactivation of Pd–Pt/YSZ and Pd–Pt/ZrO_2_ was likely due to the Pt component and not because of Pd, since Pd in the monometallic catalyst remained stable for the entire duration. The Y_2_O_3_ promotional effect on the stability of the catalysts was prominent on the tested catalysts. However, the addition of Pt to Pd/ZrO_2_ and Pd/YSZ catalyst did not inhibit the deactivation.

## Conclusions

In summary, we have demonstrated the catalytic oxidation of CH_4_ and C_2_H_4_ on Pd and Pt catalysts supported on ZrO_2_ and modified ZrO_2_ (YSZ) supports. The following conclusions are drawn from this work.

• Pd and Pt monometallic catalysts supported on ZrO_2_ and YSZ show lower light-off temperatures than bimetallic (Pd–Pt) catalysts for pure CH_4_, C_2_H_4_, and mixed CH_4_/C_2_H_4_ feed conditions.

• In the mixed hydrocarbon feed, ethylene shows a promotional effect by lowering the light-off temperature of methane.

• Pd supported on ZrO_2_ and YSZ is highly active. YSZ-supported catalysts show more resistance to water deactivation, while ZrO_2_ is severally deactivated. However, the catalytic activity of Pd/ZrO_2_ could be regenerated by a rapid temperature increase to 600 °C.

• It is noticed that the Pt-based catalysts are not deactivated in the presence of water, as the water effect starts to diminish at a temperature above 450 °C.

• Bimetallic catalysts supported on YSZ are more resistant to deactivation in wet feed gas condition than ZrO_2_-supported bimetallic catalysts. The resistance to deactivation is associated with the YSZ promotional effect rather than the Pt addition to the Pd catalyst.

• *In situ* DRIFT analysis shows OH species on the surface of both ZrO_2_- and YSZ-supported catalysts. During CH_4_ oxidation at 350 °C, however, the desorption of OH is slow on the Pd/ZrO_2_ catalysts, and accumulation is noticed on extended durations, which could be the reason for the deactivation. The steady-state accumulation of hydroxyl is recorded over the bimetallic catalysts. It might be because the water effect is small for alloy catalysts or due to the relatively low water of reaction since *T*_50_ and *T*_100_ are relatively high (400–450 °C).

Further investigation is required to optimize the Pd/Pt ratio. Many reports claim that relatively high Pt loading causes adverse effects and an increase in light-off temperature, but relatively low Pt loading has a promotional effect and causes resistance to deactivation in wet feed conditions.

## Conflicts of interest

There are no conflicts to declare.

## Supplementary Material

RA-011-D0RA10773E-s001
