# Supplementary material for: Yttrium stabilization and Pt addition to Pd/ZrO2 catalyst for the oxidation of methane in the presence of ethylene and water
Source: RSC Adv. 2021 Mar 23;11(20):11910–7. doi: 10.1039/d0ra10773e (PMC8696560; doi:10.1039/d0ra10773e)
Supplement: RA-011-D0RA10773E-s001 [file RA-011-D0RA10773E-s001.pdf]

Electronic Supplementary Material (ESI) for RSC Advances.
This journal is © The Royal Society of Chemistry 2021

Yttrium stabilization and Pt addition to Pd/ZrO_2 catalyst for the oxidation of methane in the presence of ethylene and water

Hassnain Abbas Khan*[a], Junyu Hao[a], Omar El. Tall[b] , Aamir Farooq*[a]

[a]*Clean Combustion Research Center, Physical Science and Engineering Division, King Abdullah University of Science and Technology (KAUST), Thuwal 23955-6900 Saudi Arabia.*

[b]*KAUST Core Labs, King Abdullah University of Science and Technology (KAUST), Thuwal, 23955-6900 Saudi Arabia.*

***Corresponding author email:** hassnain.khan@kaust.edu.sa, aamir.farooq@kaust.edu.sa

Details of catalyst characterization

Crystalline structures were analyzed by X-ray powder diffractometer (Bruker D2 Advance) employing Cu Kα radiation (λ = 0.15418 nm) at 30 kV and 40 mA.

Specific surface areas and pore volumes were determined by N_2 physisorption at −196 °C using Micromeritics ASAP-2020. The catalysts (0.5 g) were first degassed for 6 h at 100 °C. Specific surface area was calculated in the relative pressure (P/P_o) range of 0.01–0.2 assuming a cross-sectional area of 0.162 nm^2 for N_2. The loading of active metal component was measured by X-ray fluoresces spectroscopy.

Carbon monoxide (CO) chemisorption was performed in a pulse mode with the Micromeritics ASAP-2020 apparatus. Prior to the measurements, 0.05 g of the sample was thermally treated in a 5 % O2/He stream at 120 ℃ for 30 min to remove physically adsorbed water and other impurities. The sample was cooled down to room temperature, and heated to 300 ℃ with a heating rate of 10 ℃/min in 5% H2/Ar at a flow rate of 50 mL/min for 30 min. After the reduction, the sample was purged with He gas at the same temperature for 20 min. After cooling to ambient temperature, 10% CO/He gas was introduced for CO chemisorption. The CO loop gas was used for each pulse (a sample loop of 0.543 mL) and the pulse injections were repeated until saturation. The metal dispersion in each catalyst was calculated from the amount of CO adsorbed, assuming the stoichiometry factor (SF) for Pd/CO to be 1.0, as follows:

$$\text{Dispersion (\%)} = \frac{100 \times V_s \times S_F \times M_W}{S_W \times F_n \times 22{,}414}$$

where VS is the cumulative volume of adsorbed CO (cm3 at STP), MW is the molecular weight of Pd metal (g/mol), SW is the weight of the sample, and Fn is the Pd fraction in relation to the total weight of the catalyst sample.

Temperature-programmed desorption (O_2-TPD) analysis was carried out using Micromeritics AutoChem II 2920 apparatus. Desorbed species were analyzed by quadrupole mass spectrometry (Balzers QMS 200 Omnistar). The catalyst (0.2 g) was fixed in a quartz reactor by packing quartz wool at both ends. Prior to the measurement, the sample was oxidized in 5% O_2 in He flow (50 cm^3/min) at 800 °C for 1 h. After cooling the sample to room temperature under 5% O_2 in He flow, the feed gas was switched to pure He stream (50 cm^3/min) for O_2-TPD. The sample was ramped from room temperature to 850 °C at 10 °C/min.

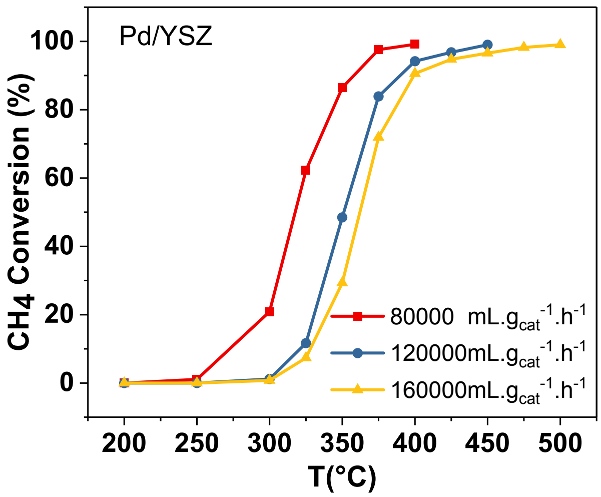

Figure S1. Dependency of Methane conversion on GHSV over Pd/YSZ catalyst.

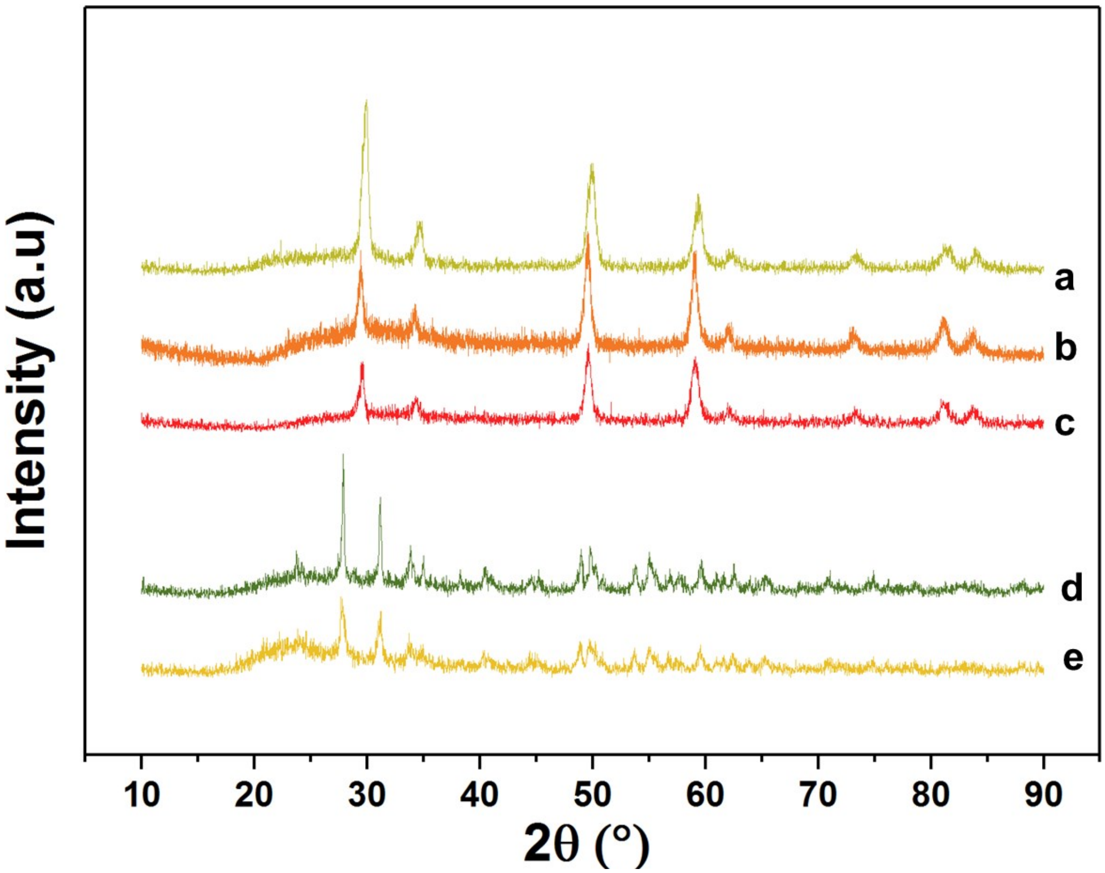

Figure S2. XRD patterns of spent catalyst after cyclic study in dry and wet conditions over the temperature range of 350-600 ^{o}C. (a) Pd/YSZ (b) Pt/YSZ (c) PdPt/YSZ (d) Pd/ZrO_2 (e) PdPt/ $ZrO_{2.}$

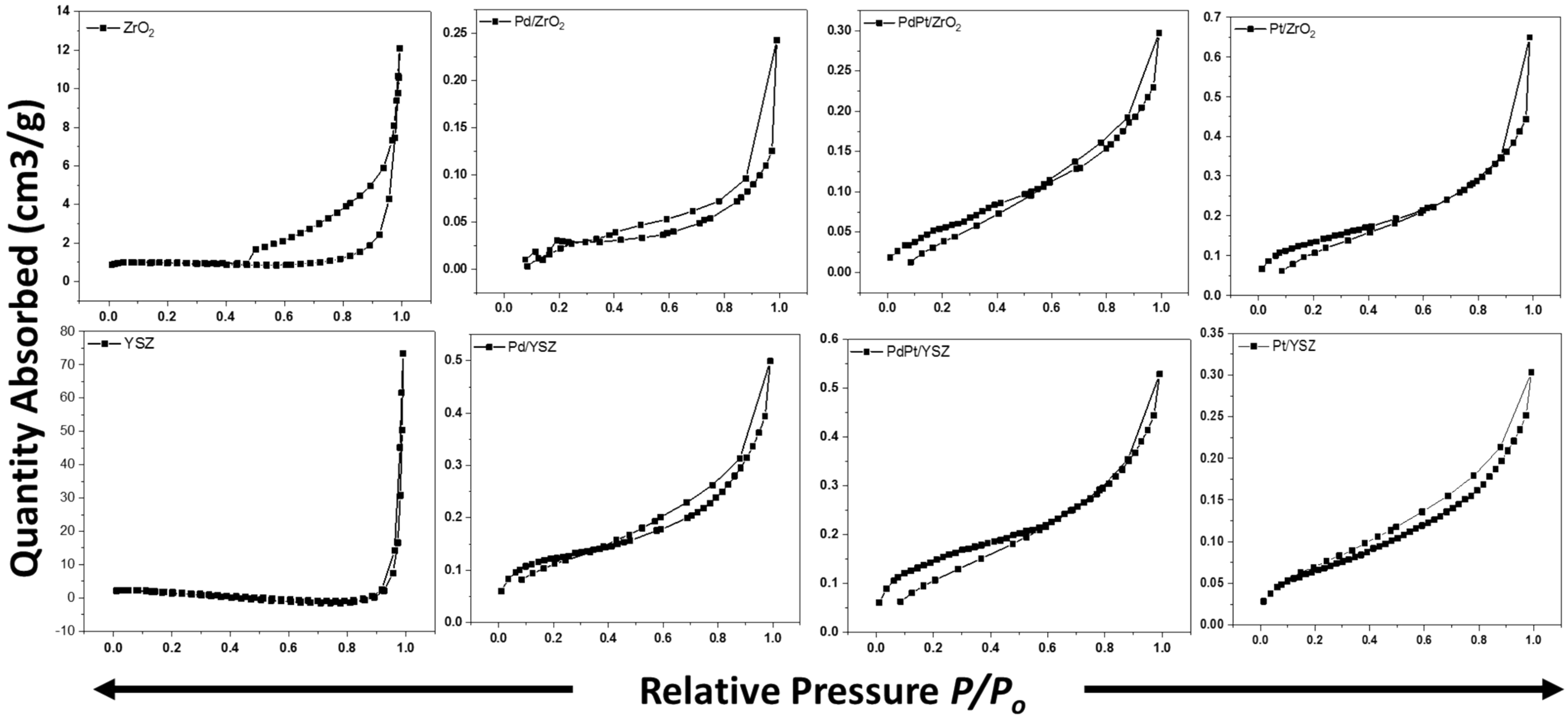

Figure S3. **N_2** Adsorption desorption isotherms of supports (ZrO_2 and YSZ) and Pd , Pt, PdPt catalysts.

Table S1. BET surface area of prepared pristine catalysts

**Catalyst**	**S_{BET} ($m^2\ g^{-1}$)**	**Pore diameter (nm)[a]**	**Average pore volume ($cm^3 g^{-1}$)**	**BET Surface by Krypton gas**
ZrO_2	3.4	41.3	1.8	5.43
YSZ	9.0	12.1	0.7	9.198
Pd/ZrO_2	2.8	24.4	0.09	2.745
Pd/YSZ	8.9	10.9	0.10	-
PtPd/ZrO_2	2.8	10.5	0.01	-
PtPd/YSZ	4.4	8.2	0.09	-
Pt/ZrO_2	4.2	8.0	0.02	-
Pt/YSZ	10.6	8.1	0.01	-
